# Clinical Effect of the Modified Morrow Septal Myectomy Procedure for Biventricular Hypertrophic Cardiomyopathy

**DOI:** 10.31083/j.rcm2501021

**Published:** 2024-01-10

**Authors:** Tong Tan, Wei Zhu, Jianrui Ma, Bingqi Fu, Xiaodong Zeng, Ruobing Wang, Xiaoyi Li, Jian Liu, Jian Zhuang, Jimei Chen, Huiming Guo

**Affiliations:** ^1^Department of Cardiovascular Surgery, Guangdong Provincial Key Laboratory of South China Structural Heart Disease, Guangdong Cardiovascular Institute, Guangdong Provincial People’s Hospital (Guangdong Academy of Medical Sciences), Southern Medical University, 510000 Guangzhou, Guangdong, China; ^2^Department of Cardiac Surgery, Beijing Anzhen Hospital, Capital Medical University, Beijing Institute of Heart, Lung and Blood Vascular Diseases, 10029 Beijing, China

**Keywords:** hypertrophic obstructive cardiomyopathy, biventricular hypertrophic cardiomyopathy, ventricular outflow tract obstruction, biventricular outflow tract obstruction, modified septal myectomy, clinical analysis, 3D printing

## Abstract

**Background:**

Right ventricular involvement in hypertrophic cardiomyopathy is uncommon. This study aimed to evaluate clinical outcomes of the modified septal myectomy in patients diagnosed with biventricular hypertrophic cardiomyopathy (BHCM), a subject seldom explored in the literature.

**Methods:**

We conducted a retrospective cohort study from January 2019 to January 2023, enrolling 12 patients with BHCM. Each patient underwent a modified septal myectomy and was followed postoperatively. Clinical data and echocardiographic parameters, including the ventricular outflow tract peak pressure gradient and maximum interventricular septum thickness, were collected and analyzed.

**Results:**

The study cohort had a median age of 43.0 (interquartile range 14.5–63.0) years at surgery, with four patients (33.3%) being children. Two patients (16.7%) previously underwent percutaneous transluminal septal myocardial ablation. Surgical relief of biventricular outflow tract obstruction (BVOTO) was achieved in five patients (41.7%), aside from those managed solely for left ventricular outflow tract obstruction. In five instances, three-dimensional (3D) printing technology assisted in surgical planning. The postoperative interventricular septum thickness was significantly reduced (21.0 mm preoperative vs. 14.5 mm postoperative, *p* < 0.001), effectively eliminating residual ventricular outflow tract obstruction. There were no severe complications, such as septal perforation or third-degree atrioventricular block. During a mean follow up of 21.2 ± 15.3 months, no sudden deaths, residual outflow tract obstruction, permanent pacemaker implantation, recurrent systolic anterior motion, or reoperations were reported.

**Conclusions:**

Our findings affirm that the modified septal myectomy remains the gold standard treatment for BHCM, improving patient symptoms and quality of life. BVOTO relief can be safely and effectively achieved through septal myectomy via transaortic and pulmonary valve approaches in selected patients. For intricate cases, the application of 3D printing technology as a preoperative planning tool is advised to optimize surgical precision and safety.

## 1. Introduction

Hypertrophic cardiomyopathy (HCM) is a genetic heart condition affecting 1:500 people and is primarily caused by mutations in genes encoding sarcomere proteins [1]. HCM has diverse phenotypic expressions resulting in clinical presentations ranging from no symptoms to sudden cardiac death (SCD). The vast majority of HCM cases are characterized by an abnormal thickness of the interventricular septum (IVS), while a few cases involve the apical and mid-segments of the left ventricle [2]. A rarer variant, biventricular hypertrophic cardiomyopathy (BHCM) may lead to the obstruction of both the left and right ventricular outflow tracts [3, 4]. Due to its rarity, surgical data on BHCM are scarce and experience in safely and effectively performing surgery for BHCM is limited. The objective of the present study was to evaluate the effectiveness of the modified septal myectomy in patients with BHCM at our center.

## 2. Materials and Methods

### 2.1 Study Population and Definitions

In this retrospective cohort study, we enrolled twelve consecutive patients with BHCM who underwent surgical treatment at our institution between January 2019 and January 2023. Eligible patients were clinically diagnosed with primary BHCM, confirmed either pathologically or through genetic testing. Patients with Noonan syndrome, Costello syndrome, aortic valve stenosis, and other diseases causing hypertrophy and ventricular outflow tract obstruction were excluded. HCM was defined as an end-diastolic left ventricular wall thickness ≥15 mm (13 mm if family history of HCM is positive) that cannot be explained by other cardiac or systemic diseases [2, 5]. Concurrently, BHCM was diagnosed if right ventricular hypertrophic cardiomyopathy was present [6], characterized by a right ventricular free wall thickness of >5 mm. Left ventricular outflow tract (LVOT) obstruction was defined as a resting or provoked systolic gradient >30 mmHg (1 mmHg = 0.133 kPa) [2, 5]. Right ventricular outflow tract (RVOT) obstruction was defined as ≥16 mmHg [7]. The probability of SCD within 5 years was calculated using a novel predictive model [8]. Patients’ health status was evaluated using the simplified Kansas City Cardiomyopathy Questionnaire [9].

### 2.2 Data Collection

Baseline characteristics, including age, gender, symptoms, personal history, and family history, were collected during the initial clinic visit. Surgical data, preoperative biological data, and electrocardiography results were retrieved from the electronic medical record system of our center. Follow-up data were acquired by outpatient review at 1, 3, 6, and 12 months postoperatively, or by telephone interviews.

Prior to surgery, all patients underwent two-dimensional echocardiographic assessments to confirm the HCM diagnosis, subtype, peak gradient at ventricular outflow tracts, and other intracardiac structural diseases, in accordance with the British Society of Echocardiography practical guidelines [10]. For complex cases requiring detailed preoperative planning, patient-specific three-dimensional (3D) reconstruction and printed models were utilized [11].

### 2.3 Surgical Procedures

All surgeries were performed under general anesthesia with cardiopulmonary bypass support. Transesophageal echocardiography was used to verify the BHCM diagnosis, evaluate hemodynamic parameters, and identify any other structural anomalies. Surgical approaches and the extent of resection were tailored for each patient based on preoperative imaging and intraoperative exploration. Extended septal myectomy in the left ventricle was performed for an LVOT peak gradient >50 mmHg at rest or with provocation. Extraseptal myectomy in the right ventricle was advised for patients with a resting RVOT peak gradient >30 mmHg. Other concomitant procedures were decided by the surgical team based on consensus guidelines and operator experience.

**Fig. 1.**
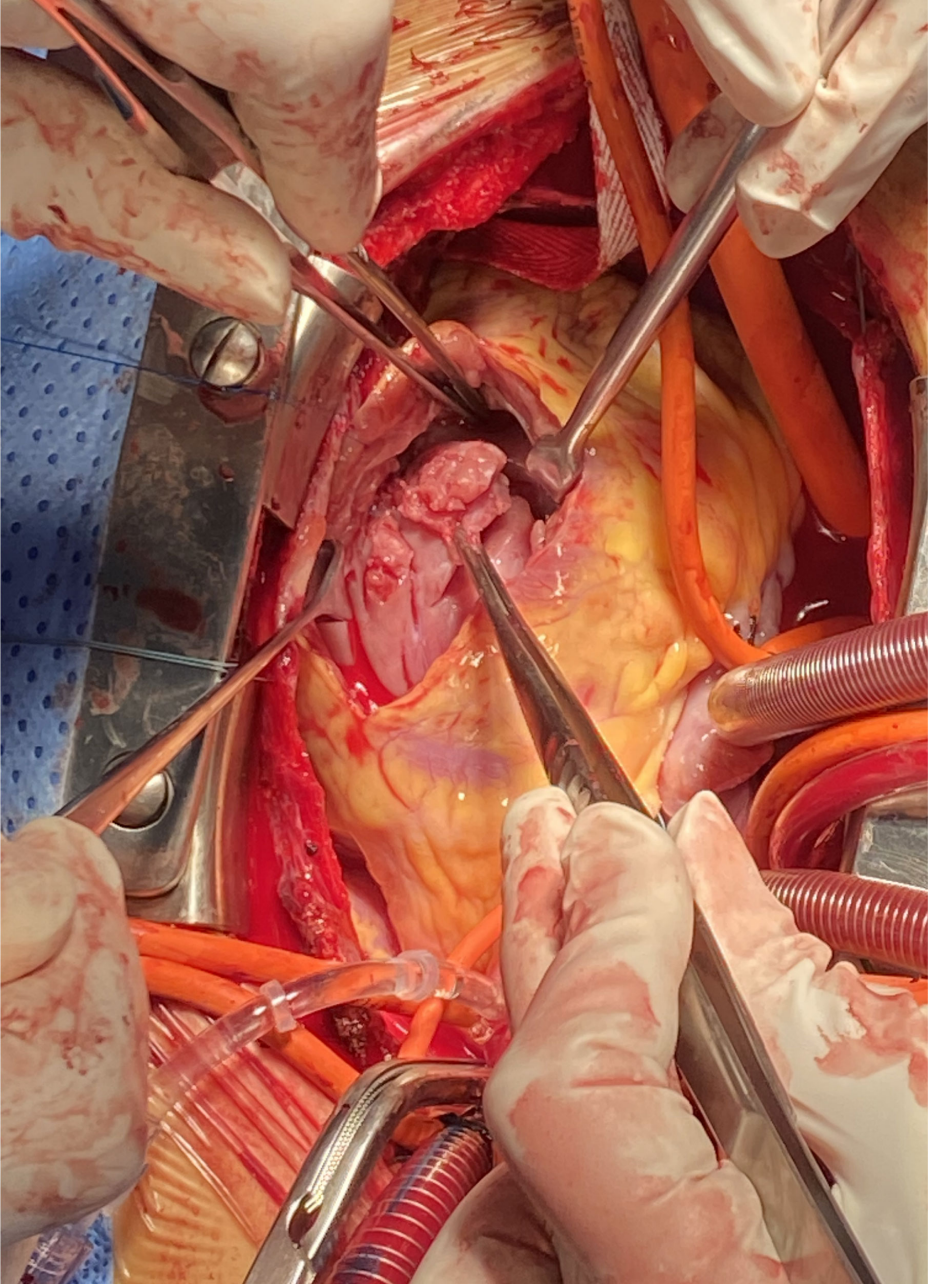
**Surgical approach for relief of right ventricular outflow tract obstruction**.

**Fig. 2.**
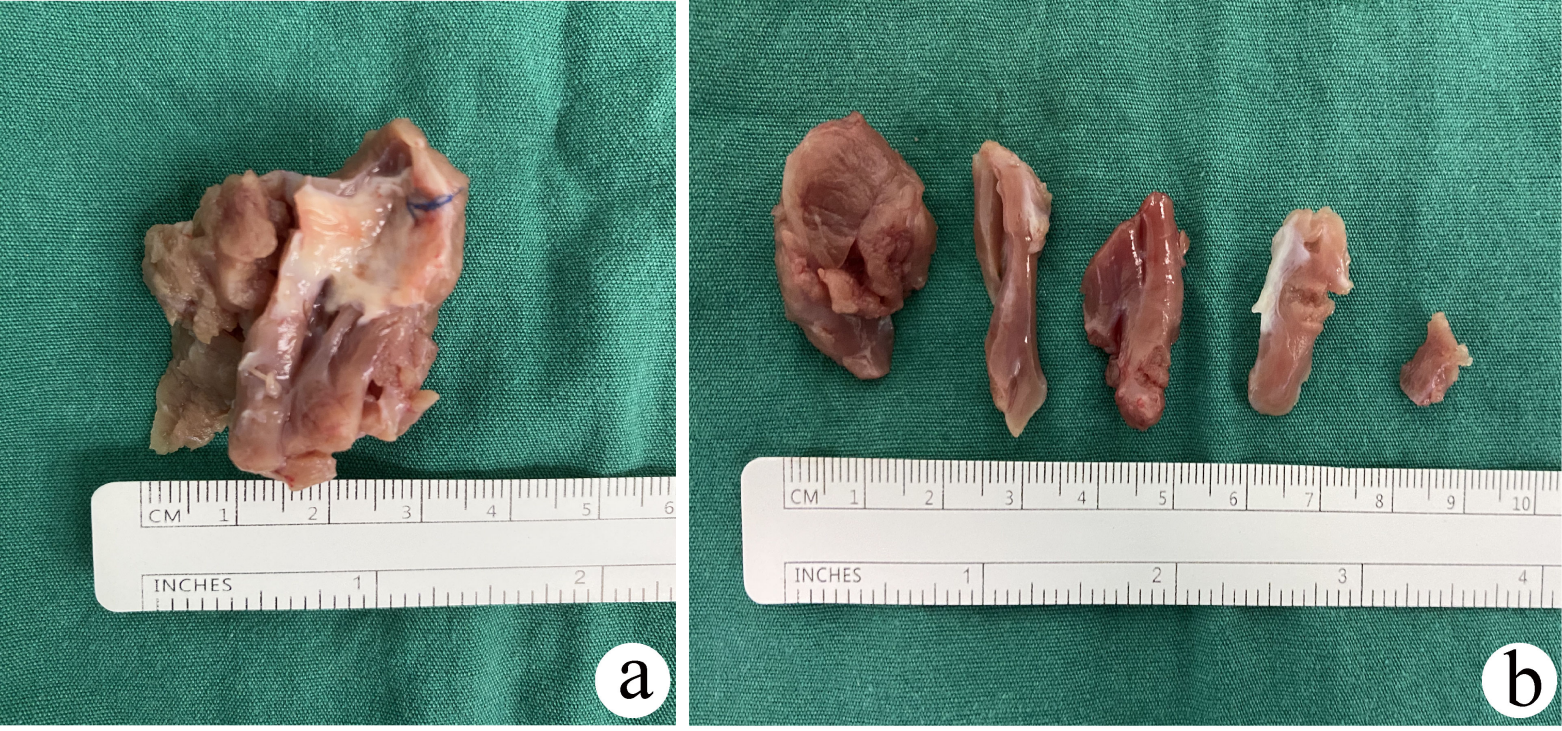
**Septum myocardium and muscle bundles resection during the procedure**. (a) From left ventricle. (b) From right ventricle.

In the first place, the extended septal myectomy was applied to relieve the LVOT obstruction. The incision started 3–5 mm to the right of the nadir of the right aortic sinus, extended leftward to the mitral anterior commissure, and downward to the apex of the left ventricle. Aberrant muscle bundles and papillary muscles were partially excised until the apex was visible through the incision. To relieve RVOT obstruction, a 3–4 cm longitudinal ventriculotomy incision was made 5–8 mm below the subpulmonary artery, away from the left anterior descending coronary artery. The IVS and right ventricle were exposed using two retractors (Fig. 1). Myocardial fibers and stiffness were detected by finger exploration. The cardiac hypertrophy located at the subpulmonary valve or conus arteriosus level was removed while avoiding damage to the subvalvular tricuspid valve apparatus. The infundibular muscle bundles were resected when they were hypertrophic, causing RVOT obstruction. If necessary, a pericardial patch with continuous 5–0 prolene sutures was used for RVOT enlargement. The resected myocardia were weighed, and the volumes were measured separately (Fig. 2).

### 2.4 Statistical Analysis

Continuous variables are presented as either medians with interquartile ranges or means ± standard deviation, depending on the Shapiro-Wilk test results. Differences between groups were assessed using the student’s *t*-test or Mann–Whitney test, as appropriate. Categorical variables are reported as frequencies (percentages) and were compared using Fisher’s exact test. All data analysis was performed using SPSS Statistics version 26.0 (SPSS Inc., Armonk, NY, USA).

## 3. Results

### 3.1 Baseline Characteristics

**Fig. 3.**
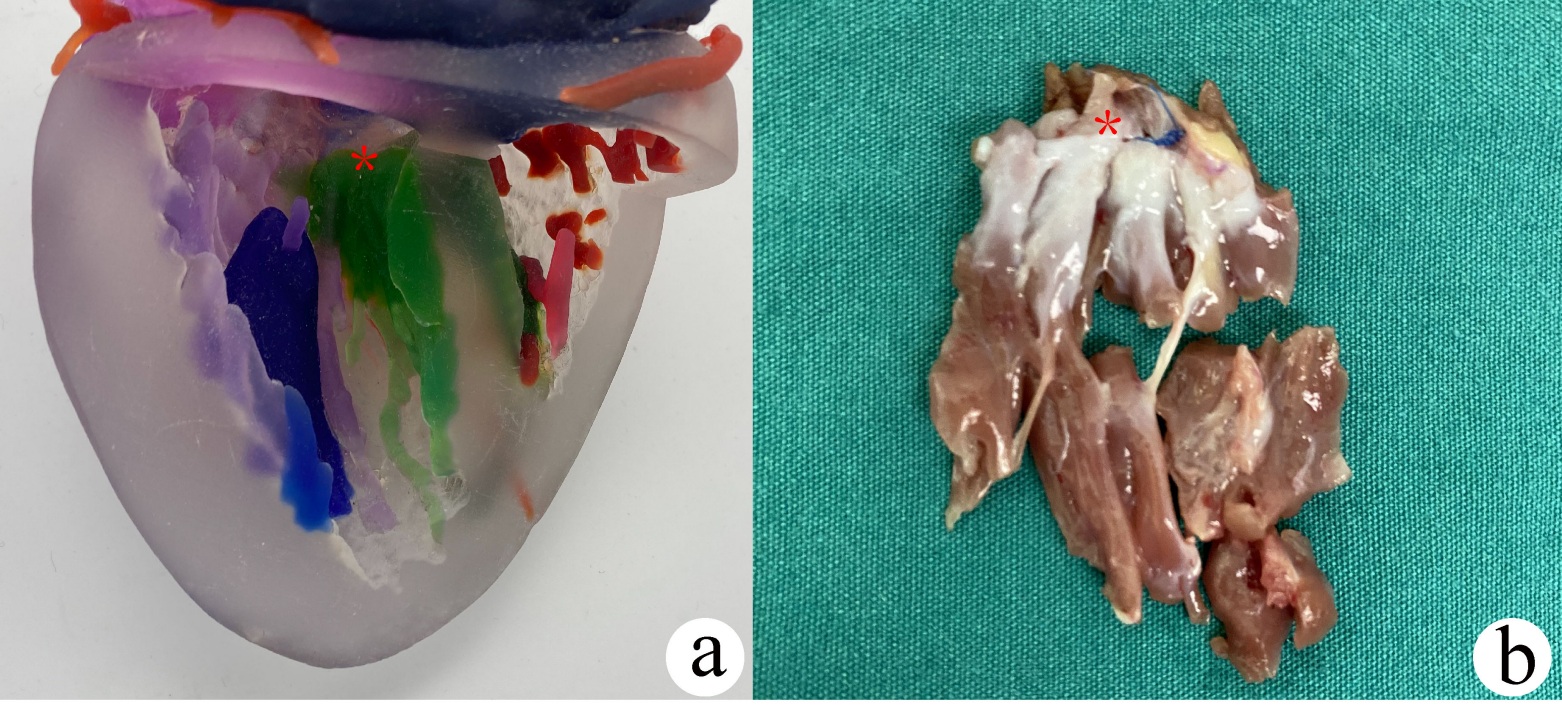
In complex cases of biventricular hypertrophic obstructive cardiomyopathy, the septum myocardium (*) was resected under the guidance of three-dimensional (3D) printing techniques. (a) An individualized 3D printing model with the myocardium that was expected to be removed (green color). (b) Actual myocardium resection.

The baseline characteristics of the 12 enrolled patients are presented in Table 1. The overall sample had a median age of 43.0 (interquartile range [IQR]: 14.5–63.0) years at surgery. Pediatric patients made up a third of the sample (4, 33.3%, exact ages: 14, 16, 14, and 12 years). Over half of the patients (7, 58.3%) were female. The average IVS thickness was 21.0 mm (IQR: 20.0–26.3 mm), with 7.0 mm (IQR: 5.8–9.1 mm) of right ventricular free wall. All patients exhibited systolic anterior motion (SAM), and showed mild-to-moderate symptoms such as syncope, shortness of breath on exertion, or chest tightness despite the maximum tolerated dose of either β-blockers or calcium channel blockers. Family history of HCM was present in two (16.7%), and two (16.7%) had previously undergone percutaneous transluminal septal myocardial ablation (PTSMA). Notably, this was the first septal myectomy for all patients. In five complex cases, the 3D printing technique was used to guide the surgery (Fig. 3).

**Table 1.** **Baseline characteristics**.

		Overall (n = 12)	BVOTO (n = 5)	LVOTO (n = 7)	*p*-value
Age, years		43.0 (14.5, 63.0)	42.0 ± 25.3	38.4 ± 23.0	0.808
Pediatric patients, n (%)		4 (100%)	3 (60.0%)	1 (14.3%)	0.222
Female, n (%)		7 (58.3%)	3 (60.0%)	4 (57.1%)	1
Family history of HCM, n (%)		2 (16.7%)	1 (20.0%)	1 (14.3%)	1
Previous PTSMA, n (%)		2 (16.7%)	1 (20.0%)	1 (14.3%)	1
SCD, %		3.0 (2.4, 3.8)	5.3 ± 4.3	3.0 ± 0.5	0.183
Pro-BNP, pg/mL		2774.2 ± 1819.3	2007.2 ± 1394.0	3322.0 ± 1982.7	0.207
Cardiac function, n (%)					1
	II	5 (41.7%)	2 (40.0%)	3 (42.9%)	
	III	6 (50.0%)	3 (60.0%)	3 (42.9%)	
	IV	1 (8.3%)	0	1 (14.3%)	
MR Grade ≥2, n (%)		8 (66.7%)	4 (80.0%)	4 (57.1%)	0.576
IVS, mm		21.0 (20.0, 26.3)	20.0 (16.5, 25.5)	22.0 (20.0, 31.5)	0.321
LVPW thickness, mm		15.1 ± 3.9	12.0 ± 2.3	17.3 ± 3.3	0.008
RVW thickness, mm		7.0 (5.8, 9.1)	6.2 (5.9, 7.2)	7.1 (5.6, 9.7)	0.328
LVOT gradient, mmHg		85.2 ± 33.1	94.6 ± 38.6	78.4 ± 29.8	0.458
RVOT gradient, mmHg		-	37.6 ± 11.9	-	-
SAM, n (%)		12 (100%)	5 (100%)	7 (100%)	-
LAD, mm		41.3 ± 7.5	43.4 ± 5.0	39.7 ± 9.0	0.387
LVEF, %		67.0 (65.0, 69.5)	66.8 ± 1.1	64.9 ± 7.0	0.495
${\mathrm{RVEF}}^{*}$, %		63.6 ± 7.1	65.6 ± 6.9	61.3 ± 7.1	0.332
RVED volume ${\mathrm{index}}^{*}$, ${\mathrm{mL/m}}^{2}$		72.3 ± 20.0	68.8 ± 12.7	75.2 ± 25.4	0.606
LVED volume ${\mathrm{index}}^{*}$, ${\mathrm{mL/m}}^{2}$		95.9 ± 17.1	83.9 ± 9.6	106.0 ± 15.6	0.020

Abbreviations: HCM, hypertrophic cardiomyopathy; PTSMA, percutaneous transluminal septal myocardial ablation; SCD, sudden cardiac death; BNP, brain natriuretic peptide; MR, mitral regurgitation; IVS, interventricular septum; LVPW, left ventricular posterior wall; RVW, right ventricular wall; LVOT, left ventricular outflow tract; RVOT, right ventricular outflow tract; SAM, systolic anterior motion; LVEF, left ventricular ejection fraction; RVEF, right ventricular ejection fraction; LAD, left atrial dimension; RVED, right ventricular end-diastolic; LVED, left ventricular end-diastolic; BVOTO, biventricular outflow tract obstruction; LVOTO, left ventricular outflow tract obstruction. ^*^These data were collected using cardiac magnetic resonance imaging in 11 patients.

### 3.2 Surgical Outcomes

All patients underwent surgical management to address left ventricular outflow tract obstruction (LVOTO). Among these, five (41.7%) patients who also achieved relief from RVOT obstruction were included in the biventricular outflow tract obstruction (BVOTO) group. In the specific case of a 14-year-old with a previous ablation procedure and RVOT gradient <30 mmHg, the biventricular obstruction was ultimately relieved. This decision was informed by concerns that irreversible septal hypertrophy could result in long-term RVOT obstruction. The modified septal myectomy effectively reduced IVS thickness (from 21.0 mm [IQR: 20.0–26.3 mm] to 14.5 mm [IQR: 11.5–19.3 mm], *p* < 0.001) without causing iatrogenic complications like septal perforation or third-degree atrioventricular blockage. The mean durations for cardiopulmonary bypass (CPB) and aortic cross clamp time were 154.5 ± 41.1 minutes and 96.0 ± 26.6 minutes, respectively.

In the LVOTO group, the mean weight of the excised myocardium was 9.6 ± 7.9 grams. Two patients underwent concomitant surgeries: one had an aortic valve replacement due to aortic regurgitation, and another underwent mitral valve repair using artificial chordae tendineae implantation. Postoperatively, the overall left ventricular outflow tract peak gradient significantly decreased from 85.2 ± 33.1 mmHg to 9.9 ± 6.2 mmHg (*p* = 0.002).

In the BVOTO group, two patients required patch enlargement, and one exhibited SAM after myectomy, necessitating a resumption of CPB for mitral valve replacement. The excised myocardial tissue weighed 7.5 ± 3.8 grams for the left ventricular side and 3.9 ± 3.5 grams for the right, significantly reducing the peak gradients for both left (from 78.4 ± 29.8 mmHg to 9.9 ± 6.2 mmHg, *p* = 0.001) and right (from 37.6 ± 11.9 mmHg to 5.2 ± 0.8 mmHg, *p* = 0.004) ventricular outflow tracts. For both groups, the median duration for intubation time was 18.6 hours (IQR: 6.7–35.3 hours), and the median intensive care unit (ICU) stay was 2.8 days (IQR: 1.7–5.7 days). No significant differences in these measures were observed between the LVOTO and BVOTO groups. Only one cardiovascular event was reported: a child in the BVOTO group developed postoperative non-sustained ventricular tachycardia, which was successfully managed by amiodarone treatment and fluid treatment. All patients were discharged without complications.

### 3.3 Follow-up 

During the follow-up period, which averaged 21.2 ± 15.3 months, no major adverse events such as sudden death, reoperation, permanent pacemaker implantation, or new-onset atrial fibrillation were observed in either the LVOTO or BVOTO groups. Two children reported chest pain, one was readmitted for medical treatment and subsequently recovered. The latest echocardiography assessment showed no recurrent ventricular outflow tract obstruction, SAM, or more than grade 2 mitral regurgitation. The overall median LVOT gradient measured 7.5 mmHg (IQR: 5.3–15.3 mmHg), and only two patients in the BVOTO group had a 3–4 mmHg gradient at the RVOT. The Kansas City Cardiomyopathy Questionnaire score (75.1 ± 8.9 preoperative vs. 84.7 ± 6.7 follow-up, *p* = 0.003) was significantly improved. In terms of functional status, six patients advanced to New York Heart Association (NYHA) class I, and four moved to class II. Only two patients remained in NYHA class III.

## 4. Discussion

While most HCM cases involve hypertrophy of the septum and left ventricle, right ventricular involvement defines a unique HCM phenotype. Previous studies [12, 13] have reported that 15%–30% of HCM patients display BHCM, which includes both left/right ventricular and biventricular obstructions. Similar to its left ventricular counterpart, right ventricular HCM features four obstruction subtypes: outflow tract (most common), inflow tract, mid-ventricle, and apex [14, 15]. Despite its clinical significance, isolated RVOT obstruction is exceedingly rare, and the epidemiology of biventricular obstruction is largely unknown, mostly documented through case reports [16, 17]. In a 10-year study by Quintana E *et al*. [18] biventricular obstruction was reported in only 0.5% (11/2283) of patients treated surgically. Biventricular obstruction appears more frequently in children and accounts for 18.8% of childhood hypertrophic obstructive cardiomyopathy [19].

While BHCM can be easily detected through echocardiography, it’s crucial to differentiate it from other hypertrophy-causing diseases or phenotypes, such as storage diseases and RASopathies [20]. It requires a complete diagnostic workup, including family history, clinical presentation, laboratory tests, detailed imaging examination, and genetic testing [21]. Establishing a diagnosis of primary BHCM is essential to ensure that medical treatment and myectomy result in a good prognosis.

Right ventricular HCM usually leads to increased ventricular stiffness and decreased ventricular wall compliance, resulting in diastolic dysfunction and impaired right heart function, therefore, BHCM patients are more likely to present with symptoms such as palpitations, fatigue, dyspnea [22]. The histological underpinnings for complications like atrial fibrillation in HCM are atrial enlargement and myocardial fibrosis [23, 24, 25]. RVOT obstruction in BHCM is associated with a higher risk of cardiovascular events and progressive heart failure [26, 27]. Long-term right heart dysfunction can exacerbate the already compromised left heart function in these patients. Therefore, to improve surgical outcomes and patient safety, it is advisable to undertake surgical intervention before the onset of severe heart failure.

The current guidelines have limited recommendations for BHCM management [2, 5]. Treatment is typically individualized, focusing on symptom relief, reducing complication risk, and preventing SCD [2, 5]. Conventional drug treatment often include β-blockers, which exert a negative inotropic effect on the myocardium, which extend ventricular filling time and improve myocardial blood supply [28]. Calcium channel blockers and disopyramide also exert negative inotropic effects.

Emerging therapies including mavacamten (MYK461) and aficamten (CK-274) have demonstrated efficacy in reducing outflow tract gradients while enhancing cardiac function and alleviating symptoms [29, 30]. Mavacamten, in particular, acts by inhibiting myosin ATPase activity and inducing a compact myosin head configuration, and has been shown to be safe and well-tolerated for various HCM phenotypes [31]. Although these developments are promising, it’s important to note that there have yet to be studies specifically evaluating the impact of these novel treatments on BHCM.

In our study, all BHCM patients exhibited an LVOTO gradient ≥50 mmHg, which remained poorly managed even with conventional drug therapy. This suggests that invasive treatments, preferably conducted in specialized centers, are warranted. Several invasive strategies are available for BHCM, including but not limited to PTSMA and septal myectomy [32]. Although ablation is less invasive, especially in children, it has some limitations. First, the success of PTSMA is dependent on the anatomy of the septal branch of the left anterior descending coronary artery, which guides the extent of induced myocardial necrosis [33]. The presence of BHCM complicates the identification of septal perforators, making it challenging to ensure effective relief of biventricular outflow tract obstruction. Overuse of ethanol also increases the risk of severe complications, such as large myocardial infarction [33]. In our cohort, two patients who had previously undergone PTSMA remained symptomatic, highlighting the potential need for reoperation in BHCM. An alternative treatment, endocardial radiofrequency ablation, targets specific areas of septal hypertrophy, but produces a minimal reduction of septal thickness [34]. Given that BHCM cases typically present with severe septal thickness, this technique has limited utility. The Liwen procedure, or percutaneous intramyocardial septal radiofrequency ablation has been introduced to address some of these limitations [35]. However, its application to BHCM remains unexplored. Consequently, the modified septal myectomy remains the gold standard for the treatment of obstructive HCM.

While our study underscores the positive outcomes of septal myectomy in selected patients with BHCM, long-term results need to be established. It’s important to note that myectomy did not completely eliminate the risk of SCD. Postoperative complications, such as non-sustained ventricular tachycardia, as observed in our study, carry a less favorable prognosis for both adult and pediatric populations [36, 37]. Consequently, ongoing risk stratification is essential during the follow-up period for BHCM patients.

When surgically addressing RVOT obstruction, the conventional “see the apex” guideline is not applicable. Given that nearly 38.8% of patients experience a left bundle branch block following left ventricle septum myectomy [38], extra caution is warranted during extensive myectomy in the right ventricle’s IVS. Combination of both left and right bundle branch block will contribute to the requirement of permanent pacemaker implantation. Borisov KV [39] suggests that myectomy should start from the right ventricle’s conal part, and avoid the moderator band. This method has shown promise in minimizing changes in ventricular penetration and lowering the risk of complete conduction block.

**Fig. 4.**
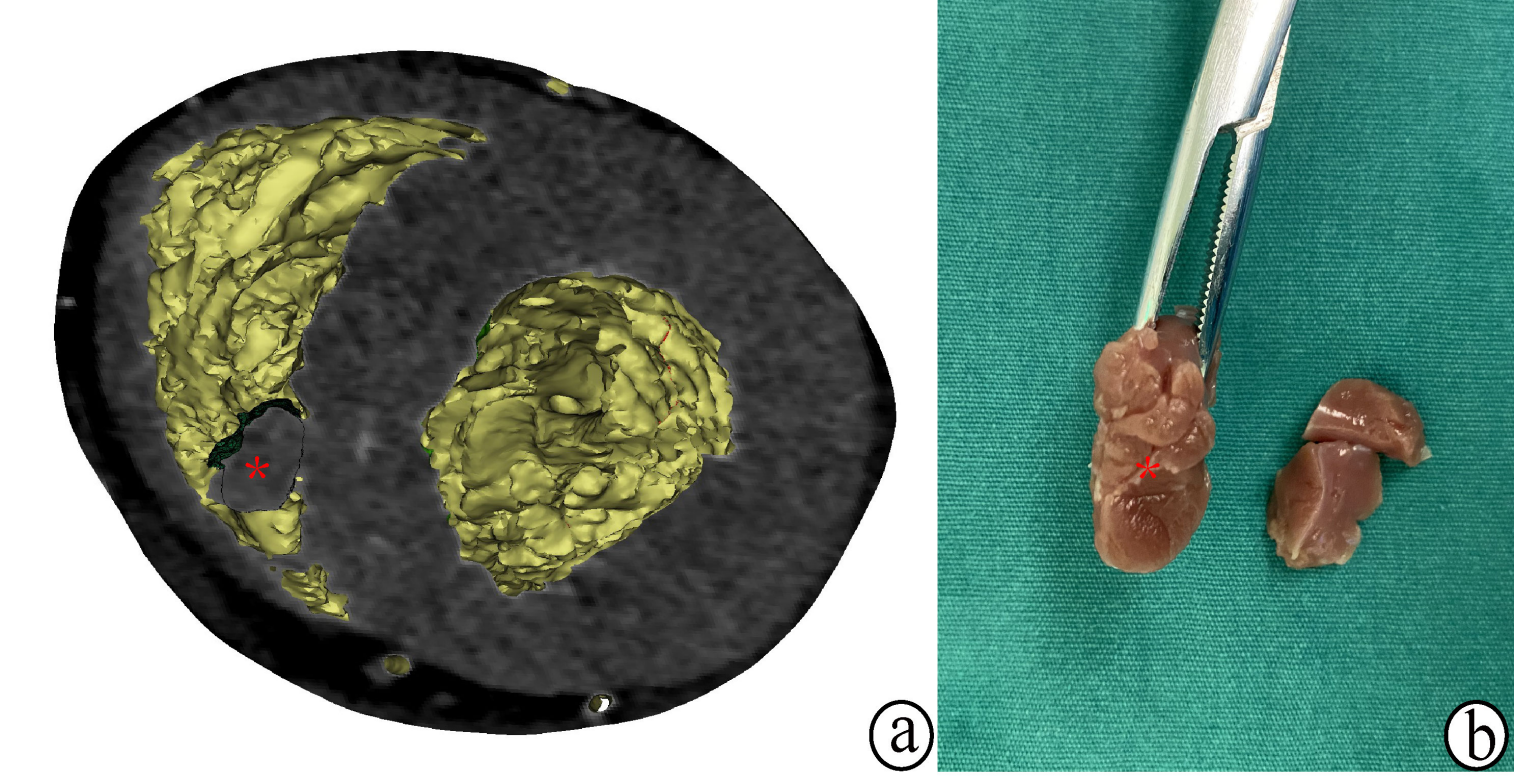
Visualization of the hypertrophic myocardium (*) in right ventricle. (a) In the three-dimensional model. (b) In the actual myocardium resection.

In our study, we found a strong correlation between RVOT obstruction and IVS hypertrophy, which contributed to the narrowing of the outflow tract. To optimize surgical planning in complex cases, we used 3D printing techniques targeting the bulging part of the hypertrophied septum in the right ventricle (Fig. 4). This preoperative tool allowed surgeons to identify and precisely measure the myocardial tissue slated for resection. Additionally, we utilized Mimics software to estimate the right ventricular cavity size, ensuring adequate myectomy in BHCM cases with significantly narrowed cavities, and providing data functionally similar to magnetic resonance imaging.

The sterilized 3D printed model served as an intraoperative guide for the myectomy procedure. Post-surgery, we compared the size and volume of the excised myocardium with the predicted measurements from the model. Leveraging the printed model enabled greater precision, minimizing the risk of severe complications such as septal perforation. Additionally, the model helped to identify other anatomical irregularities such as hypertrophied trabeculae, anomalous papillary muscles, or other subvalvular anomalies. This information proves invaluable for clinicians in deciding whether additional procedures are necessary, thereby contributing to more accurate surgical planning and potentially improved patient outcomes.

## 5. Study Limitations

This study comes with specific limitations. First, due to the rarity of BHCM, this single-center study had a limited sample size. Second, the retrospective nature of this research introduces potential biases, especially as some data—like cardiac magnetic resonance parameters—were not uniformly documented across all patients, potentially leading to a statistical bias. Finally, while our follow-up shows promising early outcomes, long-term adverse events, including late mortality, remain unknown. Future research with a larger sample size and extended follow-up would provide more robust conclusions.

## 6. Conclusions

In our cohort study, we successfully and safely alleviated BVOTO by employing septal myectomy through transaortic and pulmonary valve approaches in selected patients. These positive results reaffirm the modified septal myectomy as the gold standard in BHCM treatment, leading to symptom relief and enhanced quality of life. For complex cases, we recommend the use of 3D printing technology to guide surgical decisions and enhance surgical safety.

## Data Availability

The original contributions presented in this study are included in this article, further inquiries can be directed to the corresponding authors with appropriate reasons.
